# Maternal Pheromone Emission During Biparental Care: Evidence for Consistent Individual Differences and Links to Terminal Investment

**DOI:** 10.1007/s10886-026-01697-4

**Published:** 2026-03-23

**Authors:** Jacqueline Sahm, Cassandra Jackl, Taina Conrad, Johannes Stökl, Sandra Steiger

**Affiliations:** Department of Evolutionary Animal Ecology, University of Bayreuth, Universitätsstraße 30, 95447 Bayreuth, Germany

**Keywords:** Nicrophorus, Methyl geranate, Headspace, Family life, Terminal investment

## Abstract

Pheromone production, once considered a fixed species-specific trait, is now recognized to vary plastically within individuals and consistently among them. Such variation may reflect differences in individual quality as well as adaptive allocation strategies, for example when residual reproductive value declines and individuals exhibit terminal investment in current reproduction. While most work has focused on sex pheromones, much less is known about variation in pheromones that mediate social behavior. In the burying beetle *Nicrophorus vespilloides*, caring females emit the pheromone methyl geranate (MG), which reflects their temporary infertility during active larval care and suppresses mating behavior of the male breeding partner. Here, we examined how MG emission varies with female age and reproductive experience, whether variation in emission reflects parental investment in the current brood, and whether it shows consistent individual differences. Females reproduced once or repeatedly at four ages under standardized conditions. Age alone did not affect MG emission, but pheromone levels and parental effort increased with reproductive experience. Importantly, MG emission was positively correlated with brood and larval mass, indicating that higher pheromone production was associated with greater investment in offspring. MG emission was also repeatable across reproductive bouts. These findings suggests that MG emission exhibits both plasticity and consistent individual differences and may serve as a reliable signal of maternal investment. MG could therefore provide males not only with information about female temporary infertility but also about her level of investment in the current brood, offering a mechanism for the coordination of parental care between the sexes.

**Supplementary Information:**

The online version contains supplementary material available at 10.1007/s10886-026-01697-4.

## Introduction

Traditionally, pheromones were viewed as static signals. This is especially true for sex pheromones, which function to promote mating by eliciting attraction or courtship in conspecifics and were consequently assumed to be species-specific traits shaped by stabilizing selection. However, extensive evidence demonstrates that pheromone production can be highly variable, both between and within individuals, shaped by genetic differences as well as phenotypic plasticity (Groot et al. 2016, 2024; Pasqual et al. 2021; Steiger and Stökl 2014). While genetic variation can lead to consistent differences in pheromone quantity or composition between individuals, environmental and physiological factors can induce plastic changes, leading to variation both among individuals in different conditions and within the same individual over time (Pasqual et al. 2021).

Plastic variation in pheromone production can result from a variety of external factors - ranging from temperature (Appel and Rust 1983; Roeser-Mueller et al. 2010; Dion et al. 2016; Kárpáti et al. 2023); diet (Schultzhaus et al. 2018; Chemnitz et al. 2015; Henneken et al. 2017; Weiss and Schneider 2022; Zweerus et al. 2023), or social context (Steiger et al. 2011; Miklas et al. 2003) to internal factors such as body size (Chemnitz et al. 2015; Blaul and Ruther 2012; Buchinger et al. 2017), mating status (Kartika et al. 2020; Lu et al. 2017; Del Mazo-Cancino et al. 2004; Ayasse et al. 1999), inbreeding (van Bergen et al. 2013), immunocompetence (Burand et al. 2005; Rantala et al. 2003; Penn and Potts 1998), or age (Chemnitz et al. 2015; Nieberding et al. 2012). Among the many factors influencing pheromone production, age and physiological state play a particularly crucial role. In some cases, individuals approaching the end of their reproductive lifespan or facing survival threats have been found to exhibit shifts in pheromone production. By increasing pheromone output or altering its composition, individuals may enhance their current reproductive success when future opportunities are expected to be low and their residual reproductive value declines. Such patterns, in which reproductive effort is restrained early in life and increases as residual reproductive value declines, are consistent with the concept of terminal investment as originally formulated in life-history theory (Williams 1966; Clutton Brock 1984). With respect to chemical signaling, empirical support for terminal investment comes from systems in which residual reproductive value is reduced either by age or by external threats to survival. For example, in *Tenebrio molitor* beetles, both males and females exhibit terminal investment in pheromone production, becoming more attractive after an immune challenge or other survival threats that reduce future reproductive prospects (Nielsen and Holman 2012; Sadd et al. 2006; Mendoza-Díaz de León et al. 2024; Kivleniece et al. 2010). Similarly, other studies have shown that ageing individuals alter pheromone production in a way that enhances their attractiveness (Chemnitz et al. 2015; Cory and Schneider 2016) and in some cases also increase their calling time (Umbers et al. 2015). However, the opposite pattern has also been observed, with ageing leading to a decline in pheromone attractiveness (Kaltenpoth and Strohm 2006; Foster and Greenwood 1997; Garratt et al. 2011), potentially reflecting either senescence or late-life reproductive restraint (McNamara et al. 2009).

While most research on pheromone variation and plasticity, including work on terminal investment, has focused on sex pheromones, much less is known about how other types of pheromones vary between individuals or respond to changing conditions. In particular, pheromones involved in the coordination of social behaviors, such as parental care, are likely to show age-dependent variation. Such variation may arise either because individuals increase investment in the signals themselves later in life, or because pheromone expression reliably tracks age-related increases in parental effort and thus reflects shifts in reproductive investment as future reproductive opportunities decline. This expectation arises because such pheromones are often closely associated with the allocation of resources toward current offspring at the potential cost of future reproductive opportunities (Steiger and Stökl 2017, 2018; Mas and Kölliker 2008). Examining whether pheromones associated with parental care exhibit stable individual differences and/or plasticity in response to changing reproductive conditions could provide deeper insights into pheromone-mediated communication during parental care - a taxonomically widespread phenomenon (Clutton-Brock 1991; Royle et al. 2012). Burying beetles (genus *Nicrophorus*) are a particularly suitable model system to study this for several reasons: (i) females are known to release a pheromone, methyl geranate (MG), during brood care, which helps coordinate the allocation of resources between current and future broods (Engel et al. 2016, 2019); (ii) the beetles reproduce multiple times throughout their lifespan (Scott 1998; Hopwood et al. 2014), allowing for the examination of consistent individual differences in pheromone production over time; and (iii) they have been shown to exhibit terminal investment by allocating more resources into the current brood as the chance for future reproduction declines (Creighton et al. 2009; Benowitz et al. 2013; Billman et al. 2014; Yang et al. 2024; Farchmin et al. 2020; van Parys et al. 2024; Trumbo 2012).

Burying beetles provide elaborate biparental care for their offspring (Pukowski 1933; Eggert and Müller 1997; Scott 1998; Royle et al. 2013; Potticary et al. 2024). A beetle pair monopolizes a small vertebrate carcass, transforms it into a ball like shape, and preserves the carrion using antimicrobial secretions (Potticary et al. 2024; Körner et al. 2023; Pukowski 1933). Offspring aggregate on the carcass, where they either self-feed or parents regurgitate food to larvae (Müller et al. 1998; Smiseth et al. 2003a, b). In *N. vespilloides*, it was shown that a female-produced pheromone regulates mating and biparental care (Engel et al. 2016). At the start of a breeding event, partners mate repeatedly (Engel et al. 2014), but during active care, when larvae are young and needy, female fertility is temporarily suppressed by high titers of juvenile hormone III (JH III) in the hemolymph, a key insect hormone regulating reproductive physiology (Engel et al. 2016). Females signal this hormonal state to their male breeding partner through MG, which inhibits the male’s mating drive, thereby acting as an anti-aphrodisiac and facilitating coordinated parental care of current offspring. If larvae are removed, females resume egg-laying, stop producing MG, and males begin copulating again (Engel et al. 2014, 2016). MG emission depends on the presence of both males and larvae. Females produce MG only when a male is present (Steiger et al. 2011), and its emission increases with the number of larvae they are caring for, which aligns with a reduced likelihood of discontinuing care and producing further eggs (Engel et al. 2016; Sahm et al. 2022).

MG appears to be a key parental care pheromone, as it is also produced by other burying beetle species (Engel et al. 2019). Yet, despite its central role, it remains unclear why MG levels vary considerably among females, even when controlling for brood size and the presence of a male (Engel et al. 2016). To better understand this variation, we examined whether MG emission exhibits consistent individual differences across reproductive bouts and/or phenotypic plasticity, particularly focusing on how it responds to declining residual reproductive value with age and reproductive history. Since MG emission depends on both larval number and male presence, we controlled for these factors by providing each female with a standardized brood size and ensuring that a male partner was present during the care phase. This design allowed us to isolate variation in MG emission associated with maternal care investment per offspring, rather than differences arising from brood size per se. In the first experiment, females reproduced only once at either 20, 35, 50, or 65 days old to assess the effect of age on MG emission. Additionally, we tested whether MG levels were influenced by male copulation rate or the number of eggs laid. In the second experiment, we monitored MG emission of the same females across four reproductive bouts, ensuring that each female reproduced at all four ages (20, 35, 50, and 65 days old). Furthermore, we examined whether age and previous reproductive experience affected resource allocation to the current brood by analyzing clutch size, female weight change, and larval mass. We predicted that the amount of MG emitted would increase with female age, reflecting increased allocation to the current brood as residual reproductive value declines. We further expected this age-related increase in the amount of MG to be stronger when females reproduced multiple times, as the combination of age and accumulated reproductive costs should lead to a greater decline in residual reproductive value than age alone.

## Methods and Materials

### Origin and Husbandry of Beetles

We investigated outbred *Nicrophorus vespilloides* beetles originating from wild-caught beetles collected in a deciduous forest in Bayreuth, Germany. Beetles were housed individually in plastic boxes (12 × 9 × 4.5 cm) containing moist peat and kept in a climate chamber under a 16:8 h dark: light cycle at 20 °C. Beetles were fed twice a week with sliced mealworms (*Tenebrio molitor*).

### Experiment 1: Effect of Age, Copulation Rate, and Clutch Size on MG Levels of Breeding Females

**Fig. 1:**
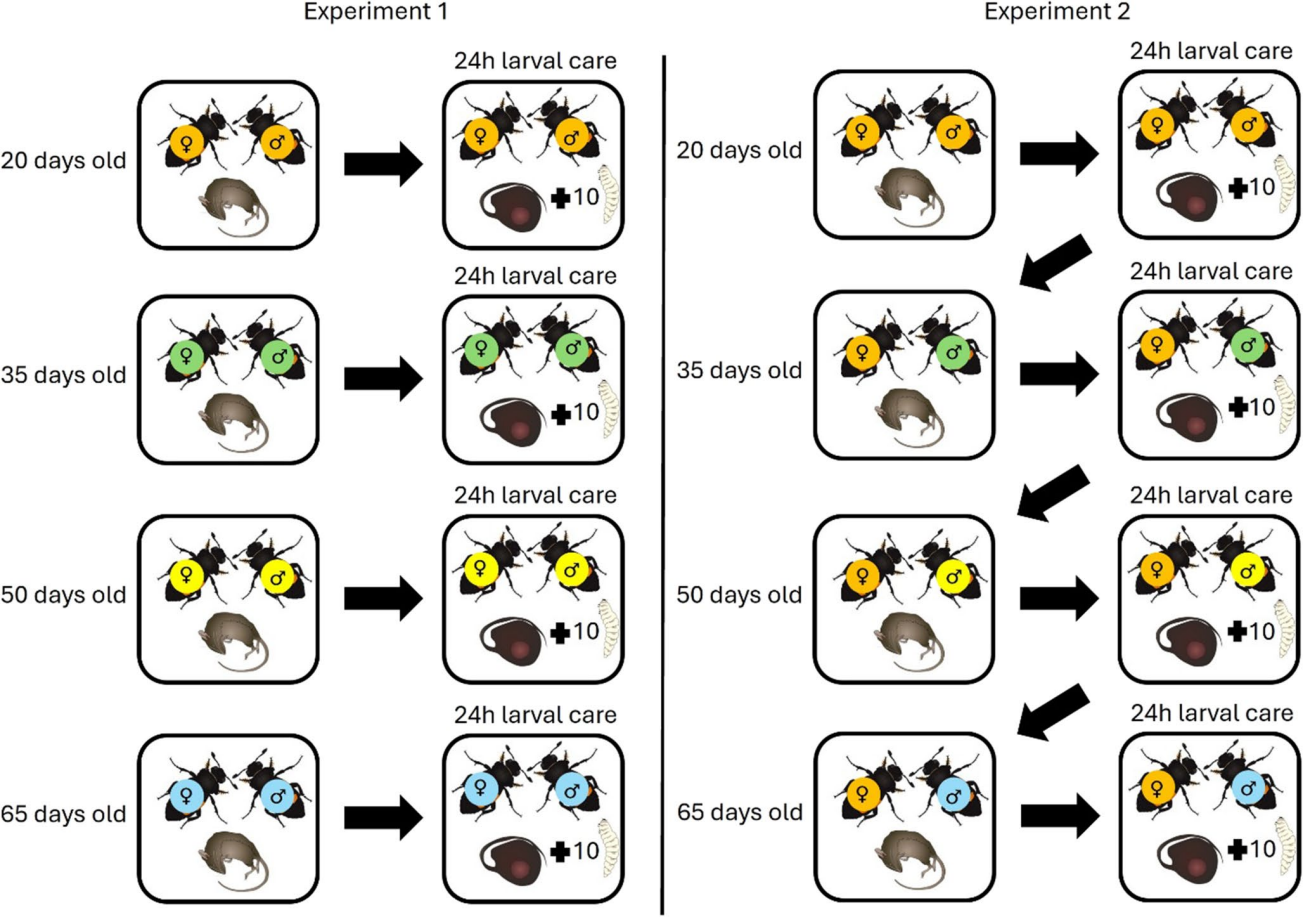
Schematic overview of the experimental designs of Experiments 1 and 2. In Experiment 1, females of different ages (indicated by different colors) were allowed to care for larvae together with a similar aged male partners (indicated by different colors) for 24 h before their pheromone emission was measured. In Experiment 2, the same females (indicated by the same color) were used across four reproductive bouts (every 15 days), each time caring for larvae with a different male partner (indicated by different colors) for 24 h before pheromone emission was measured

The first experiment investigated the effect of female age, copulation rate, and clutch size on the amount of MG emitted by breeding females. To this end, we set up 24 pairs in four different age categories (20, 35, 50 or 65 days) across two blocks using two follow-up generations (Fig. 1). Some pairs failed to reproduce or produced eggs that did not hatch, resulting in the following sample sizes for each age category: N_20 days_ = 20, N_35 days_ = 18, N_50 days_ = 24, N_65 days_ = 21. We began by haphazardly pairing unrelated virgin females and males in plastic boxes (12 × 9 × 4.5 cm) lined with moist paper tissue. Before the behavioral observations, we measured the pronotum width of each beetle using a slide gauge as a proxy for body size. Additionally, we weighed the females prior to reproduction. To assess mating behavior, pairs were allowed to acclimate for 10 min, after which we recorded the number of successful copulations during a 30-minute observation period. We monitored up to four boxes simultaneously in 2-hour intervals. Copulation rate was assessed prior to carcass introduction because quantifying mating during carcass preparation and egg laying is logistically demanding, as individuals bury the carcass and frequently move under and around it, as well as into the surrounding soil, with mating events spread over extended periods (Engel et al. 2014). Assessing copulation under standardized conditions before access to a carcass follows approaches used in previous studies of burying beetles (Hopwood et al. 2016; Head et al. 2014). Following the mating observations, each beetle pair was transferred to a similarly sized plastic box half-filled with moist peat and provided with a pre-weighed mouse carcass (approx. 10 g) to initiate breeding. After 48 h, we transferred each pair along with their carcass to a new box filled with moist peat to separate the eggs from the parents and allow the larvae to hatch in isolation. This is a well-established procedure (Eggert et al. 2025; Sahm et al. 2023; Capodeanu-Nägler et al. 2018) that allowed us to manipulate brood size in subsequent steps. We then counted the eggs left behind in the original boxes to determine each pair’s clutch size. Twenty-four hours later (i.e. 72 h after the beetles were given access to a carcass), we checked the original boxes every two hours for hatched larvae. Newly hatched larvae were pooled in a Petri dish lined with moist tissue to prevent desiccation. We then randomly assigned 10 larvae of mixed parentage to each beetle pair, but only if the pair’s own larvae had already hatched. This is a common procedure in burying beetle research (Engel et al. 2016; Oldekop et al. 2007; Sahm et al. 2022; Rauter and Moore 1999), as *N. vespilloides* beetles cannot distinguish between their own and unrelated larvae once their own offspring have hatched (Müller and Eggert 1990). A brood size of 10 larvae lies within the natural range for the carcass size used and was chosen to match the brood size used in the experiment examining MG emission in Engel et al. (2016). After 24 h of parental care, we weighed the females and sampled the emitted volatiles of females using headspace sampling (see details below) before freeze-killing all beetles at -20 °C. We collected MG after 24 h of parental care since Engel et al. (2016) found the highest MG production at this point in *N. vespilloides*.

### Experiment 2: Female MG Emission across Four Reproductive Bouts

The second experiment investigated whether MG emission is repeatable within females and influenced by the number of reproductive bouts. To this end, we measured MG emission in females during parental care across four consecutive breeding events (at 20, 35, 50, and 65 days of age), with each female sampled during all four bouts (Fig. 1). Because MG is used by females to signal to males, we paired females with a different male each time to assess the repeatability of MG emission independently of male identity and to avoid confounding effects of repeated partner interactions. We measured MG emission in a total of 11 females that successfully reproduced in all four reproductive bouts.

Breeding was initiated in the same way as in the first experiment, using pre-weighed mouse carcass (approx. 10 g) placed in a transparent box (12 × 9 × 4.5 cm) filled with moist peat. Prior to carcass provisioning, we measured the pronotum width and body weight of each female. The subsequent steps - transferring pairs after 48 h to separate eggs from parents, determining clutch size, monitoring for larval hatching, pooling newly hatched larvae, and assigning 10 larvae of mixed parentage to each pair - followed the same protocol as described above. Consistent with the first experiment, we weighed females and collected female-emitted MG using headspace sampling 24 h after the onset of parental care (see details below). In contrast to the first experiment, however, we did not freeze-kill the females but counted the larvae and measured the mass of the entire brood. Afterwards larvae and males were removed, and females were placed singly into a new box filled with moist peat and were fed with mealworms until the next reproductive bout. We measured total brood mass, average larval mass, and female weight change as proxies for female investment, assuming that heavier larvae and greater maternal weight loss reflect higher investment.

### Headspace Sampling

To analyze the amount of MG emitted by breeding females, we collected their emitted volatiles via headspace sampling. Headspace samples were collected in a climate chamber at 20 °C. Each female was individually placed in a silanized glass jar (3 cm inner diameter). Similar to previous experiments (Engel et al. 2016; Engel et al. 2019; Sahm et al. 2024), the glass jar was equipped with a headspace filter, a membrane pump, and an activated charcoal filter (50 mg; Supelco, PA, USA) to purify incoming air. The headspace filters comprised a 2 cm-long glass tube (inner diameter: 2 mm) sealed at both ends with silanized glass wool (Sigma-Aldrich, Supelco, St. Louis, USA) and filled with 3 mg of Carbotrap^®^ B and 3 mg of Tenax TA^®^ (both from Sigma-Aldrich, Supelco, St. Louis, USA) as adsorbents. Prior to use, the filters were conditioned using a Clean Cube (SIM, V1.0, Oberhausen, Germany). Volatiles of females were accumulated for 20 min before we created an airflow through the instrument for 5 min using the membrane pump (~ 200 ml/min). The headspace filters, containing the absorbed volatiles, were then stored in a freezer at -20 °C. Before analysis, we added 1 µl of methyl undecanoate (= 20 ng; Sigma-Aldrich, St. Louis, USA) dissolved in *n*-hexane (Carl ROTH, ROTISOLV^®^ ≥ 99%; Karlsruhe, Germany) as internal standard. The volaties were desorbed at a temperature of 300 °C for 8 min using a thermal desorber (Shimadzu, TD-30R) coupled to GC-MS (Shimadzu GC2030 gas-chromatograph connected to a Shimadzu QP2020NX mass-spectrometer; Shimadzu, Duisburg, Germany). The GC was equipped with a non-polar capillary column (SH-Rxi-5Sil MS, length = 30 m, inner diameter = 0.25 mm, film thickness = 0.25 μm, Shimadzu, Duisburg, Germany). The oven temperature within the GC was programmed to increase from 50 °C to 200 °C at a rate of 5 °C/min. Subsequently, the temperature was raised to 280 °C at a rate of 15 °C/min and held at this level for 10 min. Helium served as the carrier gas (linear velocity = 36.3 cm/sec). We calculated the absolute amount of MG emitted by dividing the peak area of MG by the peak area of the internal standard and multiplying it by the known quantity of the internal standard.

### Statistical Analyses

We analyzed and plotted all data using R version 4.2.2. In the first experiment, we analyzed the effect of female age, copulation rate (number of successful copulations divided by the observation time of 30 min), clutch size and female size on the amount of MG emitted using a generalized linear model (GLM) fitted with a Gaussian error structure. Model fit was assessed using the simulateResiduals() function of the *DHARMa* package (Hartig 2024). Because residuals indicated deviations from uniformity, the amount of MG was log₁₀-transformed prior to analysis. Next, we tested whether reproductive investment varied with female age by analyzing clutch size and copulation rate using GLMs fitted with Poisson error structures, and relative female weight change using a Gaussian GLM. Female age and body size were included as predictors in all models, and clutch size was additionally included in the model of relative female weight change, as females laying more eggs are expected to experience greater weight loss during reproduction. Relative weight change was calculated by dividing the difference between the female’s weight prior to reproduction and her weight at the time of MG measurement by her weight prior to reproduction. All reported F-, χ²- and p-values were obtained using the Anova()-function of the *car* package (Fox and Weisberg 2017).

In the second experiment, we tested whether MG emission of females varied with reproductive experience by fitting a generalized linear mixed model (GLMM) with MG emission as the response variable and reproductive bout number and female size as fixed effects. The model was fitted with a Gaussian error structure using the glmer() function from the *lme4* package (Bates et al. 2015). In a separate set of GLMMs, we examined how reproductive investment changed across reproductive bouts by modelling average larval mass, total brood mass, and relative female weight change (all with Gaussian error structures), as well as clutch size (with a Poisson error structure), as response variables. In these models, reproductive bout number was included as a fixed effect. Finally, to test for associations between MG emission and reproductive investment, we fitted GLMMs with MG emission as the response variable and average larval mass, total brood mass, relative female weight change, or clutch size included as fixed effects in separate models. In all GLMMs, female identity was included as random factor to account for repeated measure. Additionally, we analyzed the repeatability of MG emission, total brood mass, average larval mass, relative female weight change and clutch size across reproductive bouts using the rptGaussian() function from the *rptR* and *statmod* packages (Stoffel et al. 2017).

In both experiments, we conducted post hoc pairwise comparisons for all significant effects using the glht() function with Tukey-method. Full post hoc test results are provided in the Electronic Supplementary Material.

## Results

### Experiment 1: Effect of Age, Copulation Rate, Clutch Size on MG Levels of Breeding Females

**Fig. 2:**
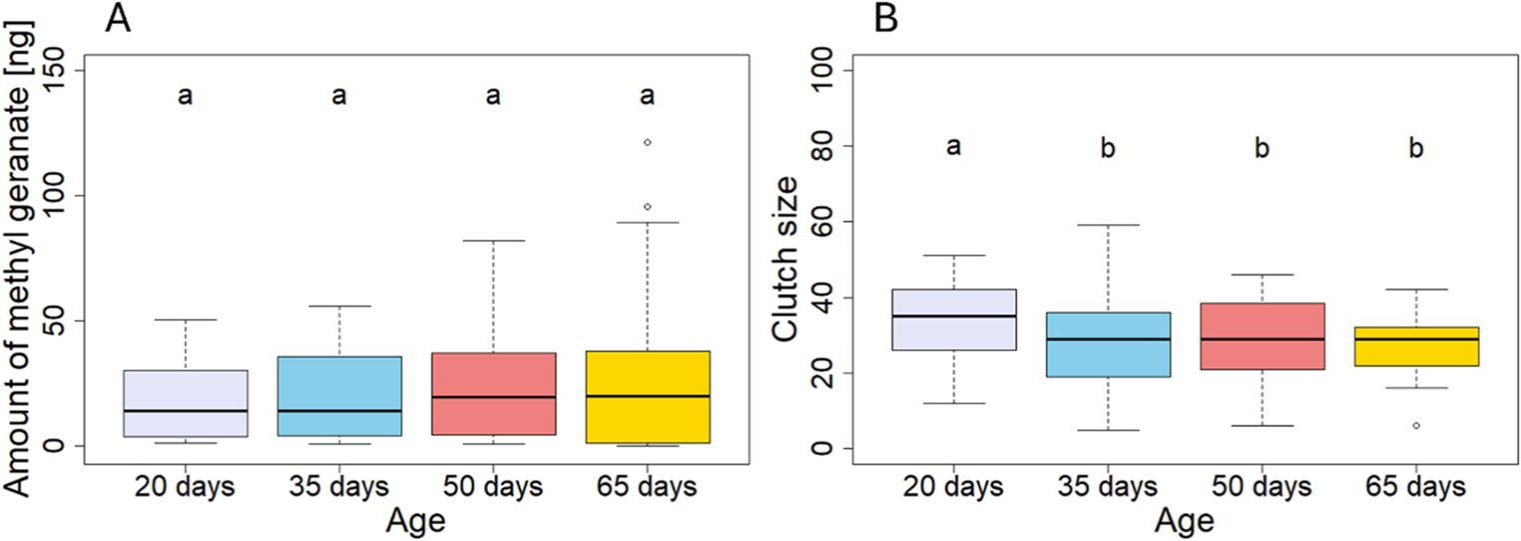
**A**) Amount of methyl geranate [ng], and **B**) clutch size (numbers of eggs laid) of females of different ages. The ages (days after eclosion) refer to the time when reproduction was induced. The boxplots illustrate the median and interquartile range, with whiskers extending to the most extreme data points within 1.5 times the interquartile range

Female age had no effect on the amount of MG emitted (*GLM*, F_3,76_ = 0.62, *P* = 0.89, Fig. 2A). Similarly, neither copulation rate (*GLM*, F_1,76_ = 0.006, *P* = 0.94) nor clutch size (*GLM*, F_1,76_ = 0.008, *P* = 0.93) influenced the amount of MG. Female body size also had no effect on MG emission (*GLM*, F_1,76_ = 0.88, *P* = 0.35) nor on clutch size (*GLM*; χ²_1,78_ = 2.91, *P* = 0.09). We then tested whether resource allocation varied with age, using copulation rate, clutch size, and female weight change as proxies. None of these traits varied significantly with female age (copulation rate: *GLM*, F_3,78_ = 1.11, *P* = 0.77; weight change: *GLM*, χ²_3,77_ = 2.76, *P* = 0.70), with the exception of clutch size, which was highest in the youngest females and significantly reduced at later ages (*GLM*, χ²_3,78 =_ 18.51, *P* = 0.0003; Fig. 2B, Table S1). Interestingly, female body size was associated with copulation rate, as larger females engaged in more copulations than smaller ones (*GLM*, χ²_1,78_ = 4.99, *P* = 0.03; Fig. S1). Female body size and clutch size had no effect on relative female weight change (clutch size: *GLM*, χ^2^_1,77_ = 0.4, *p* = 0.53; female body size: *GLM*, χ^2^_1,77_ = 0.02, *p* = 0.9).

### Experiment 2: Female MG Emission Across Four Reproductive Bouts

**Fig. 3:**
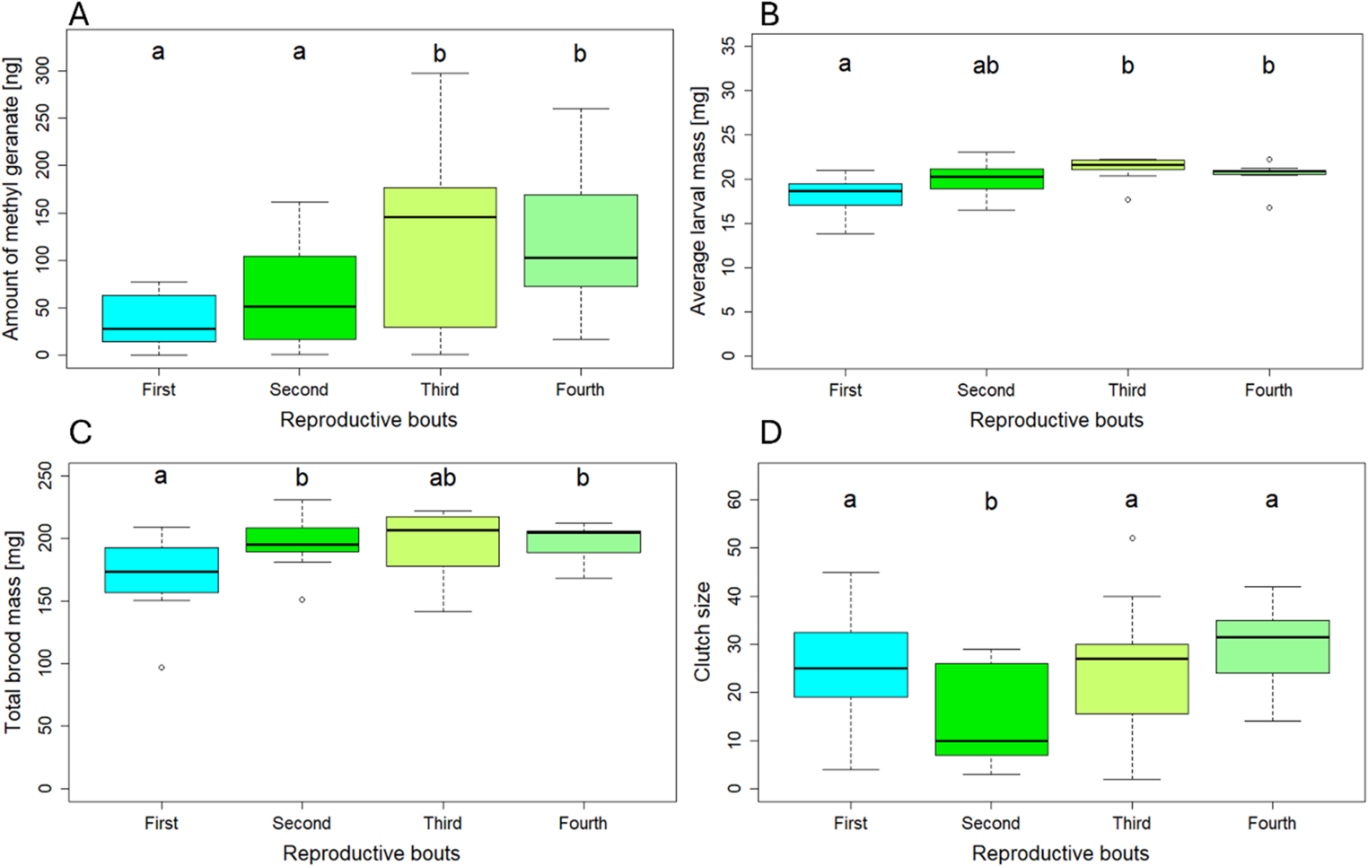
**A**) Amount of methyl geranate [ng], **B**) average larval mass [mg] after 24 h of parental care, **C**) total brood mass [mg], and **D**) clutch size (number of eggs laid) across four consecutive reproductive bouts (N = 11). Boxplots illustrate the median and interquartile range, with whiskers extending to the most extreme data points within 1.5 times the interquartile range

Reproductive bout number significantly affected female MG emission, with MG levels increasing with successive reproductive bouts and then plateauing (*GLMM*, χ²_3_ = 23.25, *P* = 0.001; Fig. 3A, Table S2). A similar pattern was observed for female investment in the current brood: both average larval mass (*GLMM*, χ²_3_ = 16.9, *P* = 0.0007; Fig. 3B, Table S3) and total brood mass (*GLMM*, χ²_3_ = 10.18, *P* = 0.02; Fig. 3C, Table S4). Clutch size was also significantly affected by reproductive bout (*GLMM*, χ²_3_ = 41.38, *P* < 0.0001; Fig. 3D, Table S5), though the pattern was non-linear with a transient reduction in clutch size during the second reproductive bout, while clutch sizes in the first, third, and fourth bouts were similar. In contrast, the relative female weight change was not affected across reproductive bouts (*GLMM*, χ²_3_ = 6.18, *P* = 0.1; Fig. S2). We next tested whether MG emission reflects female investment. MG emission correlated with both total brood mass (*GLMM*, χ²_1_ = 4.70, *P* = 0.03) and average larval mass (*GLMM*, χ²_1_ = 7.87, *P* = 0.005), with females caring for heavier broods and heavier larvae releasing higher amounts of MG. In contrast, there was no evidence of a correlation between MG levels and either relative female weight change (*GLMM*, χ²_1_ = 0.04, *p* = 0.85) or clutch size (*GLMM*, χ²_1_ = 0.12, *P* = 0.72). Our analyses further revealed that MG levels were repeatable across reproductive bouts (*R* = 0.52, *P* = 0.003), suggesting consistent individual differences in signaling. In contrast, neither total brood mass (*R* = 0.1, *P* = 0.27), average larval mass (*R* = 0.07, *P* = 0.34), relative female weight change (*R* = 0.05, *P* = 0.4), nor clutch size (*R* = 0.11; *P* = 0.22) were repeatable across reproductive bouts. To exclude the possibility that this repeatability in MG was simply due to larger females consistently emitting higher amounts than smaller ones, we tested whether body size affected MG emission. However, as in the first experiment, body size had no effect on MG emission (*GLMM*, χ²_1_ = 0.70, *P* = 0.40).

## Discussion

MG is an important pheromone in burying beetles, emitted by caring females, reflecting her hormonal state and temporarily infertility during active care and acting as anti-aphrodisiac (Engel et al. 2016, 2019). Here, we studied variation in MG emission of breeding *N. vespilloides* females, examining both plastic changes across age and reproductive bouts as well as consistent individual differences. Age alone had no effect on MG emission, but levels rose with reproductive experience, which naturally covaries with age. Investment in offspring also increased across reproductive bouts, resulting in heavier larvae and broods. These findings suggest that both advancing age and accumulated reproductive costs contribute to declining reproductive value and a terminal investment strategy, with MG emission reliably reflecting this increased parental investment. Consistent with this interpretation, females that produced more MG also invested more into their offspring, rearing heavier larvae than those with lower emission. Finally, MG levels were repeatable within females across multiple reproductive bouts, indicating that MG emission also reflects consistent individual differences.

Our first two key findings were that age alone had no effect on maternal MG levels, but age in combination with reproductive experience did, with MG levels increasing up to the third bout and then plateauing. Previous studies have shown that age can induce terminal investment in parental care and can also alter male sex pheromone emission in burying beetles. According to the “dynamic threshold model” of Duffield et al. (2018), such age-related changes are expected only once cues of reduced future reproduction exceed the threshold required to trigger increased current reproductive effort, and this threshold may itself vary with intrinsic condition or across species. Although an age of approx. 60 days, as in our study, has been shown to elicit terminal investment in caring female *N. orbicollis* (Creighton et al. 2009) and to alter sex pheromone emission in *male* *N. vespilloides* (Chemnitz et al. 2017), our study focused on *female* *N. vespilloides*, and these females may not yet have been old enough to reach the relevant threshold. Indeed, females of this species can live for more than 200 days under laboratory conditions (Steiger et al. 2012). In line with the threshold model, we found that a combination of reproductive experience and age (reproductive experience necessarily covaries with age in our study) resulted in increased MG emission, suggesting that cumulative reproductive costs together with ageing may have pushed females closer to the threshold at which reduced residual reproductive value triggers terminal investment. Importantly, life-history theory emphasizes that future performance is constrained not by chronological age per se, but by state-dependent deterioration resulting from the accumulation of damage over time (McNamara et al. 2009; McNamara and Houston 1996). Because damage accumulation is expected to depend on activity, reproductive effort may be a key driver of state deterioration, such that reproductive experience, rather than age alone, determines when the threshold for terminal investment is reached. Consistent with this state-dependent threshold view, similar patterns have been found in another burying beetle species (Farchmin et al. 2020) and in crickets (Duffield et al. 2018), where an immune challenge triggered terminal investment only in older individuals, indicating that certain cues are insufficient on their own but become effective once accumulated costs have already reduced residual reproductive value.

That MG varies not just with reproductive bout but indeed reflects greater reproductive investment when residual reproductive value is reduced is further supported by our third key finding: older reproductive experienced females produced broods of higher total mass and raised heavier larvae than younger and inexperienced females. Although we did not directly observe female behavior in this study, this pattern is likely explained by higher feeding effort in such females, as feeding has been shown to enhance offspring growth in burying beetles (Eggert et al. 1998, 2025; Rauter and Moore 2002; Capodeanu-Nägler et al. 2016). Excitingly, while our earlier work showed that elevated MG emission reflected temporary infertility - i.e. a suppression of future reproduction (Engel et al. 2016) - the present study suggests that MG also reflects the extent of care devoted to the current brood, as MG levels directly correlated with brood mass and average larval mass. Taken together, these results are consistent with the idea that MG reliably reflects parental investment as defined by Trivers (1972), simultaneously indicating increased investment to current offspring and reduced capacity for future reproduction.

In principle, however, an alternative causal interpretation cannot be excluded. Rather than reliably reflecting female parental investment, females might terminally invest directly in MG production, with elevated MG emission eliciting a stronger anti-aphrodisiac response in males, thereby redirecting male effort away from mating and toward care of the current brood. Under this scenario, higher brood mass would arise indirectly through increased male parental care rather than increased maternal investment. Nevertheless, findings from earlier experimental work support the interpretation that MG emission tracks the caregiving context and female investment. In particular, experimental manipulation of brood size has been shown to increase MG emission by females (Engel et al. 2016). Because larger broods typically impose a higher parental workload, this response suggests that MG emission reflects caregiving demands and female parental effort.

In addition to age and reproductive experience, variation in nutritional history could also influence parental investment and MG emission across breeding attempts. Repeated access to vertebrate carrion could, in principle, improve female condition and thereby facilitate greater investment in subsequent broods. However, all females in our experiment had access to carrion prior to MG measurement and larval mass assessment, including during their first reproductive attempt. Moreover, parental investment in burying beetles does not increase monotonically with cumulative nutritional intake; research has shown that prior reproductive experience can override simple nutritional effects, with females investing more following reproduction on lower-quality resources than after breeding on higher-quality resources (Billman et al. 2014). Together, these findings suggest that while nutritional history may modulate individual condition, the coordinated increase in MG emission, brood mass, and average larval mass observed here is unlikely to be driven by nutrition alone and is more consistent with age- and experience-dependent changes in reproductive investment.

Interestingly, both MG levels and average offspring mass increased from the first to the third reproductive bout, but did not increase further thereafter. The relatively early increase in investment may reflect the ecology of burying beetles, in which access to suitable carcasses is rare and difficult to monopolize, making future reproductive opportunities uncertain even after only a few successful breeding attempts and favoring increased investment once reproduction is secured. The subsequent plateau may reflect an upper physiological limit to investment per offspring; because brood size was held constant in our experimental design, total reproductive investment could not increase once this limit was reached. Alternatively, increasing senescence with age may offset any tendency toward greater terminal investment in later reproductive bouts.

Our previous work showed that MG emission is directed toward males and suppresses their copulation attempts, which is expected since females producing high amounts of MG do not lay further eggs (Engel et al. 2016). The present results, however, suggest an additional layer of signaling: MG levels also indicate how much a female invests in her current brood. This raises the intriguing possibility that males may perceive MG not only as a signal of female temporary infertility during care but also as information about her level of parental care. Such a mechanism could provide a means for the coordination of biparental care. Because biparental care involves sexual conflict over parental investment (Trivers 1972; Lessells 2012), coordination between parents may arise through negotiation (McNamara et al. 1999), whereby parents adjust their care in response to their partner’s behavior to reach a stable balance of investment. Indeed, our own work (Körner et al. 2026) and that of others (Smiseth et al. 2005; Lambert and Smiseth 2024) has shown that males tend to increase their feeding rate when females feed less, and vice versa. MG may therefore function as a chemical signal mediating the complementary adjustment of parental effort between the sexes. If MG influences male care decisions in this way, it would provide a mechanistic basis for the negotiation of parental effort between the sexes to solve sexual conflict (McNamara et al. 1999), a process that is widely observed across biparental species but whose underlying mechanisms are often unknown (Harrison et al. 2009) and which could be mediated by MG in burying beetles. While this interpretation remains hypothetical, it generates a clear prediction: future studies should test whether experimental manipulation of maternal MG emission alters male feeding rate.

A further important finding was that MG showed consistent individual differences: some females consistently emitted higher amounts of MG across bouts, whereas others emitted less. This repeatability cannot simply be explained by female body size, which remains stable over time, because body size had no effect on MG emission in either experiment. It also cannot be attributed to male identity, as males were replaced in each bout and females were always paired with a different partner. Consistent individual differences in pheromone emission have also been reported in other systems, for example in the sex pheromone of burying beetle males (Chemnitz et al. 2017), as well as in male butterflies (Nieberding et al. 2012) and female moths (Blankers et al. 2021). In these cases, repeatability has often been interpreted as reflecting variation in signaler quality under sexual selection. Although MG is not a sex pheromone, a similar explanation may apply here, with females in better condition working at a higher rate and consistently producing higher amounts of MG. If this were the case, one might expect brood mass or average larval mass to also be repeatable. However, neither trait showed consistent individual differences. A possible explanation is that males adjust their feeding effort in response to female behavior, thereby compensating for differences among females. Such male adjustments may mask potential repeatability in brood traits that would otherwise result from consistent differences in female care.

In conclusion, our study showed that maternal pheromone emission exhibits both plastic variation across reproductive bouts and consistent individual differences. Moreover, because MG levels were positively correlated with brood mass and average larval mass, MG may reliably reflect maternal investment and could provide males with information about the extent of female care, thereby facilitating coordination of parenting between the sexes. Future studies should investigate whether MG emission correlates with female feeding rate and whether maternal MG also influences male effort. 

## Supplementary Information

Below is the link to the electronic supplementary material.

Supplementary Material 1 (DOCX 49.7 KB)

## Data Availability

Data will be submitted to DRYAD.
