# Supplementary material for: Maternal Pheromone Emission During Biparental Care: Evidence for Consistent Individual Differences and Links to Terminal Investment
Source: J Chem Ecol. 2026 Mar 23;52(2):30. doi: 10.1007/s10886-026-01697-4 (PMC13009076; doi:10.1007/s10886-026-01697-4)
Supplement: Supplementary file 1 — Supplementary Material 1 (DOCX 49.7 KB) [file 10886_2026_1697_MOESM1_ESM.docx]

**Electronic Supplementary Material**

**Maternal Pheromone Emission During Biparental Care: Evidence for Consistent Individual Differences and Links to Terminal Investment**

Jacqueline Sahm^1^*, Cassandra Jackl^1^, Taina Conrad^1^, Johannes Stökl^1^, Sandra Steiger^1^

^1^ Department of Evolutionary Animal Ecology, University of Bayreuth, Universitätsstraße 30, 95447 Bayreuth, Germany

*Correspondence author: Jacqueline.Sahm@uni-bayreuth.de

**Supplementary Tables**

**Table S1:** Post hoc pairwise comparisons for the effect of female age (in days) on clutch size (number of eggs laid).

| **Response** | **Predictor** | **estimate (±SE)** | **ratio** | **p-value** |
| --- | --- | --- | --- | --- |
| **Clutch size** | age |  |  |  |
|  | 35 vs. 20 | -0.18 (0.06) | -3.04 | **0.01** |
|  | 50 vs. 20 | -0.19 (0.05) | -3.45 | **0.003** |
|  | 65 vs. 20 | -0.22 (0.06) | -3.75 | **<0.001** |
|  | 50 vs. 35 | -0.01 (0.06) | -0.16 | 0.998 |
|  | 65 vs. 35 | -0.04 (0.06) | -0.62 | 0.93 |
|  | 65 vs. 50 | -0.03 (0.06) | -0.5 | 0.96 |

**Table S2:** Post hoc pairwise comparisons for the effect of reproductive bouts on the absolute amount of methyl geranate produced by females.

| **Response** | **Predictor** | **estimate (±SE)** | **ratio** | **p-value** |
| --- | --- | --- | --- | --- |
| **MG** | Reproductive bouts |  |  |  |
|  | 2 vs. 1 | 22.15 (22.22) | 0.997 | 0.75 |
|  | 3 vs. 1 | 83.3 (21.42) | 3.89 | **<0.001** |
|  | 4 vs. 1 | 84.94 (24.79) | 3.43 | **0.003** |
|  | 3 vs. 2 | 23.1 (2.65) | 2.65 | **0.04** |
|  | 4 vs. 2 | 22.57 (2.78) | 2.78 | **0.03** |
|  | 4 vs. 3 | 25.74 (0.06) | 0.06 | 1 |

**Table S3:** Post hoc pairwise comparisons for the effect of reproductive bouts on the average larval weight.

| **Response** | **Predictor** | **estimate (±SE)** | **ratio** | **p-value** |
| --- | --- | --- | --- | --- |
| **Average larval weight** | Reproductive bouts |  |  |  |
|  | 2 vs. 1 | 1.81 (0.78) | 2.32 | 0.09 |
|  | 3 vs. 1 | 3.00 (0.77) | 3.88 | **<0.001** |
|  | 4 vs. 1 | 2.39 (0.81) | 2.95 | **0.02** |
|  | 3 vs. 2 | 1.19 (0.8) | 1.49 | 0.44 |
|  | 4 vs. 2 | 0.58 (0.82) | 0.71 | 0.89 |
|  | 4 vs. 3 | -0.61 (0.83) | -0.74 | 0.88 |

**Table S4:** Post hoc pairwise comparisons for the effect of reproductive bouts on the total brood mass.

| **Response** | **Predictor** | **estimate (±SE)** | **ratio** | **p-value** |
| --- | --- | --- | --- | --- |
| **Total brood mass** | Reproductive bouts |  |  |  |
|  | 2 vs. 1 | 27.01 (10.46) | 2.58 | **0.048** |
|  | 3 vs. 1 | 25.8 (10.32) | 2.5 | 0.06 |
|  | 4 vs. 1 | 28.06 (10.85) | 2.59 | **0.048** |
|  | 3 vs. 2 | -1.21 (10.75) | -0.11 | 1 |
|  | 4 vs. 2 | 1.04 (10.92) | 0.1 | 1 |
|  | 4 vs. 3 | 2.25 (11.14) | 0.2 | 1 |

**Table S5:** Post hoc pairwise comparisons for the effect of reproductive bouts on clutch size (number of eggs laid).

| **Response** | **Predictor** | **estimate (±SE)** | **ratio** | **p-value** |
| --- | --- | --- | --- | --- |
| **Clutch size** | Reproductive bouts |  |  |  |
|  | 2 vs. 1 | -0.52 (0.11) | -4.66 | **<0.001** |
|  | 3 vs. 1 | -0.14 (0.09) | -1.58 | 0.39 |
|  | 4 vs. 1 | 0.1 (0.1) | 0.95 | 0.77 |
|  | 3 vs. 2 | 0.37 (0.11) | 3.32 | **0.005** |
|  | 4 vs. 2 | 0.62 (0.1) | 6.11 | **<0.001** |
|  | 4 vs. 3 | 0.24 (0.11) | 2.32 | 0.09 |

**Supplementary Figures**


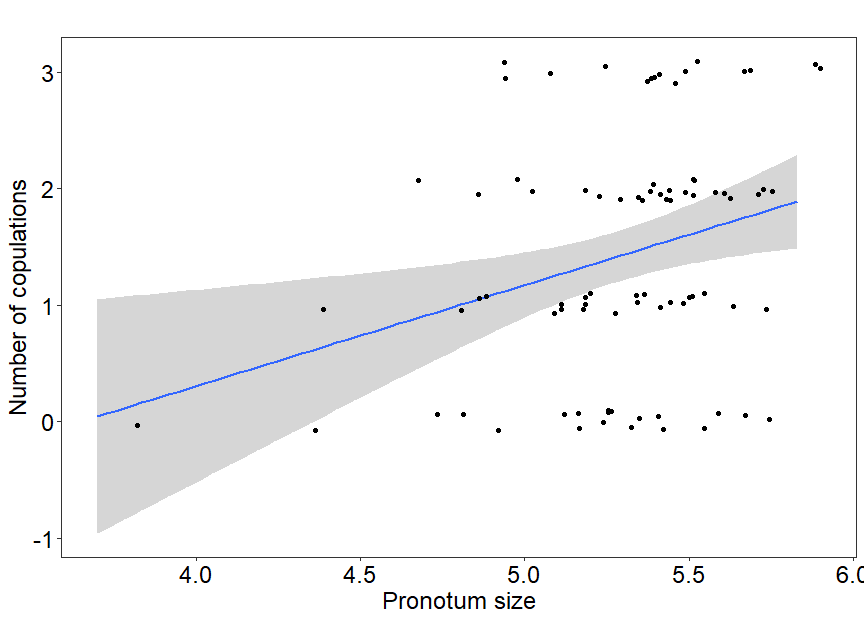


**Figure S1:** Relationship between the pronotum size of females and the number of copulations observed in 30 minutes (N = 83). The dots represent the original data, the line represents the calculated regression line and their respective 95% CI.


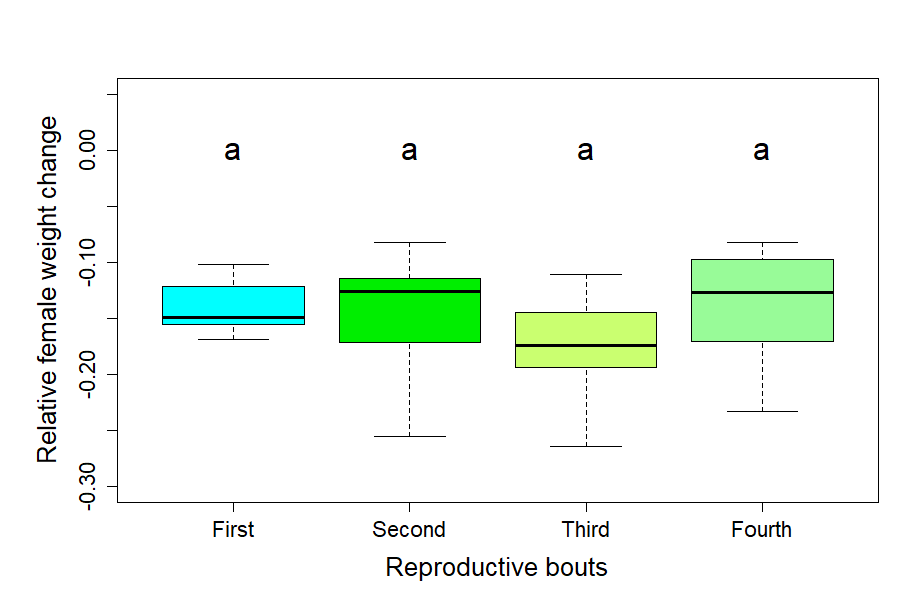


**Figure S2**: Female weight change across four consecutive reproductive bouts (N = 11). Boxplots illustrate the median and interquartile range, with whiskers extending to the most extreme data points within 1.5 times the interquartile range.
